# Effects of auxetic shoe on lumbar spine kinematics and kinetics during gait and drop vertical jump by a combined in vivo and modeling investigation

**DOI:** 10.1038/s41598-022-21540-6

**Published:** 2022-10-31

**Authors:** M. Rahmani Dehaghani, Amir Nourani, N. Arjmand

**Affiliations:** Department of Mechanical Engineering, Sharif University of Technology, Tehran, 11155-9567 Iran

**Keywords:** Biomedical engineering, Mechanical engineering

## Abstract

The present study examined the effects of auxetic shoes on the biomechanics of the spine, as compared to barefoot and conventional shoe conditions, during gait and drop vertical jump (DVJ) activities using a combined in vivo and musculoskeletal modeling approach. Motion and force-plate data as well as electromyographic (EMG) activities of select trunk muscles of 11 individuals were collected during foregoing activities. In DVJ activity, two main phases of first landing (FL) and second landing (SL) were studied. In the FL phase of DVJ noticeable alternations were observed when auxetic shoes were used. That is, compared to the conventional footwear condition, smaller EMG activities in extensor muscles (by ~ 16–29%, *p* < 0.001), smaller anterior–posterior (AP) distance between the center of pressure of ground reaction force and heel (by ~ 19%, *p* = 0.002), generally larger maximal hip, knee, and ankle flexion angles (*p* < 0.005) and finally smaller maximal L5-S1 compression force and maximal external moment (by ~ 12 and 8%, respectively, *p* < 0.001) were obtained by wearing auxetic shoes. Our results, therefore, indicate that using auxetic shoes can reduce load on the lumbar spine during high-demanding activities such as vertical jump and thus may decrease the musculoskeletal risk of injuries during these activities.

## Introduction

During daily, occupational, or recreational activities, the human spine undergoes considerable mechanical loads whose magnitude depends on body kinematics that, in turn, may be affected by the type of wearing shoes. For instance, wearing forefoot off-loader shoes (FOS) in upright standing indicates immediate biomechanical alterations such as significant increases in pelvic obliquity, pelvic torsion, lateral deviation and surface rotation as compared to conventional shoes^1^. Also, using the FOS during walking has been reported to increase pelvic obliquity and lateral deviation of the spine. The effects of wearing rocker sole shoes on center of pressure (CoP) of ground reaction force (GRF) have been examined^2^; i.e., in the anteroposterior (AP) direction, an increase in the mean value of root mean square error of CoP displacement ($CoP_{RMSE}$) (6.41 (2.97) mm) and mean CoP velocity ($CoP_{VEL}$) (4.10 (2.97) mm) is observed compared to barefoot condition. However, long-term use of rocker-sole shoes does not appear to influence postural stability in people with chronic low back pain. Using unstable shoes has also been found to cause an immediate decrease in the variability of frontal-plane foot CoP offset, transverse-plane ankle moment, and frontal-plane shoulder angle in comparison to stable shoes^3^. However, the transverse-plane spine angle variability has been reported to increase during walking in the unstable configuration. Such alterations in body segment kinematics caused by the type of wearing shoes may potentially affect spine kinetics and loadings.

Drop vertical jump (DVJ) activities have commonly been used to analyze the effect of wearing shoes on different biomechanical parameters such as vertical ground reaction force (vGRF)^4–6^, muscle activations^7–9^ and kinematics of the lower extremities^9,10^. For instance, wearing shoe, as compared to barefoot conditions, has been found to generate significantly larger ankle joint angles at initial ground contact, smaller knee joint angles between the second peak and take-off as well as smaller foot strike angles at both initial ground contact and take-off during DVJ activities^9^. The effect of shoe midsole hardness on lower extremity biomechanics during DVJ indicates that shoes with a softer midsole can cause higher forefoot peak forces but lower rearfoot peak forces, lower peak flexion moments at the ankle and hip joints, and greater prelanding muscle activations in the rectus femoris and tibialis anterior^11^.

Some types of shoes have also been found to cause changes in body dynamics response; e.g., running shoes with rounded soles (i.e., Masai Barefoot Technology, Switzerland) reduce ankle joint moments and GRF peaks in the sagittal plane compared to flat sole conventional shoes^12^. The variations in the kinematic and kinetic parameters at the knee and hip have, however, been found insignificant when performing heavy barbell back squat experiments in three different footwear conditions; i.e., barefoot, running shoes, and weightlifting shoes^13^. In this latter study, lumbosacral joint compression and shear loads have been reported to be slightly affected by the type of squatting footwear. A kind of shoes whose likely effects on spinal kinematics and kinetics remain to be investigated is auxetic shoes. Auxetic foams, in which the tension in one direction results in extension in one or more transverse directions^14^, have been recently used in shock absorber pads and personal protective equipment because of their improved conformability and superior energy absorption^15^. Due to these properties, shoes with auxetic midsoles have, therefore, been released^16^.

The present combined in vivo and musculoskeletal modeling study aims to investigate the effect of auxetic running shoes on the biomechanics of the human spine when compared to non-auxetic running shoes and bear-foot conditions during gait as well as DVJ activities. More specifically, we aim to determine whether wearing auxetic shoes affects vGRF, CoPs, spine kinematics, electromyographic (EMG) activities of select trunk muscles and/or lumbosacral (L5-S1) compressive and shear loads. Gait and DVJ experiments are performed by 11 healthy male individuals and GRFs, CoPs, and EMG activities of select muscles are measured. Subsequently, L5-S1 compressive and shear loads are estimated using a detailed full body lumbar spine (FBLS) musculoskeletal model. Therefore, in vivo data are used to either validate the musculoskeletal model (e.g., EMG data) or as input into the subject-specific musculoskeletal model (e.g., kinematics of the joints/markers). Moreover, some in vivo data (e.g., CoP, GRF, and kinematics) are separately analyzed to investigate the effect of footwear condition on spine kinetics and kinematics. Based on the proven effects of shoe soles on the biomechanics of joints^9,11–13^ as well as the higher energy absorption capacity of auxetic materials/structures (due to their higher flexural deformation as compared to conventional/bare footwear conditions) during impact loading^15,16^, it is hypothesized that wearing auxetic shoes alters spine kinematics and thus its kinetics at least during demanding (i.e., DVJ) activities.

## Material and methods

The study included both in vivo data collection and musculoskeletal modeling that are described in details below.

### in vivo study

**Figure 1:**
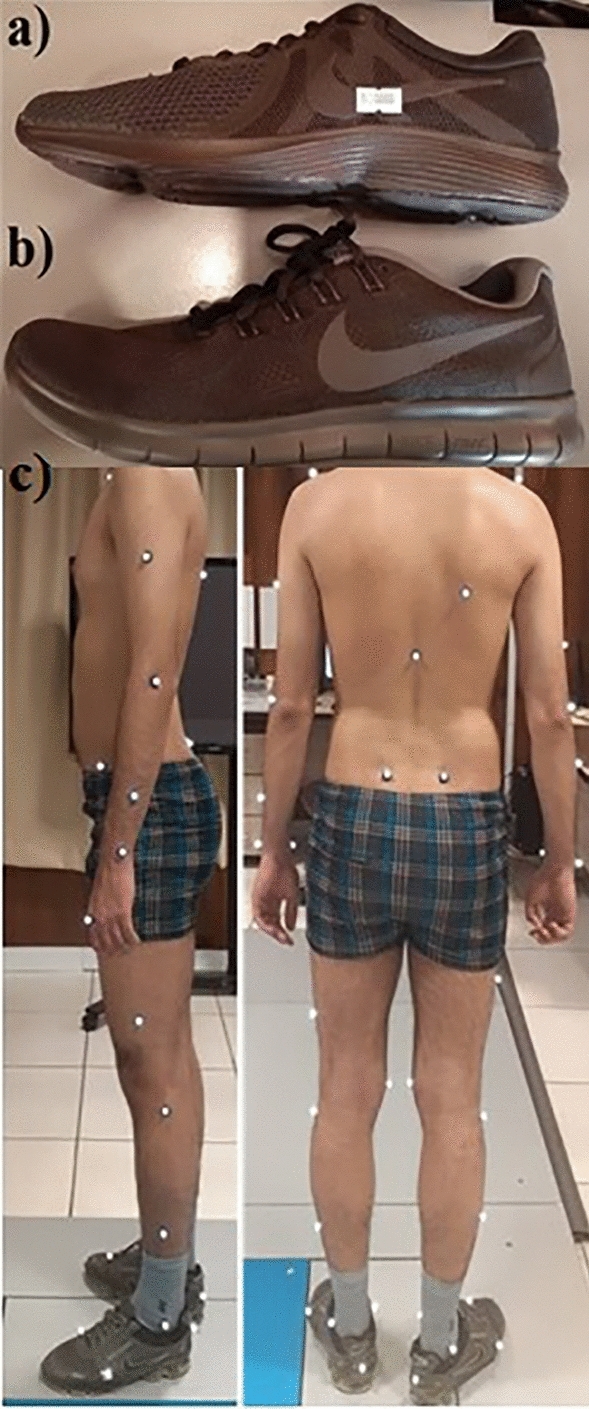
(**a**) Conventional running shoe, (**b**) auxetic Nike free RN shoe and (**c**) placement of Vicon markers in the sagittal and frontal planes.

Motion, force-plate, and EMG data were collected from 11 healthy individuals during gait and DVJ activities with three different footwear conditions: barefoot, conventional running shoes (Fig. 1a), and auxetic Free RN running shoes (Fig. 1b). Men’s shoes with European standard size of 43 were used as footwear in this study. The experiments were performed in Mowafaghian Research Centre of Intelligent Neuro-Rehabilitation Technologies (Tehran, Iran).

#### Participants

Eleven healthy young normal-weight male volunteers (23.6 ± 0.5 year-old, 175.3 ± 5.3 cm, 76.9 ± 9.9 kg, and BMI = 22.3 ± 1.2 kg/m^2^) with no recent back/knee/hip pain participated in the study. The study was approved by Tehran University of Medical Sciences Research Ethics Board (Approval No. IR.TUMS.SPH.REC.1397.252). An informed consent is obtained from all participants. All methods were carried out in accordance with relevant guidelines and regulations. Shoes were normally worn by the subjects neither being tight nor loose. Care was taken for the subjects to wear the shoes with the same protocol regarding shoelaces tying.

#### Motion analysis

A 10-camera Vicon motion capture system (Vicon Motion Systems Inc., Oxford, UK) was used at a sampling frequency of 120 Hz. According to Vicon Plug-in-Gait marker placement and using double-sided tapes, thirty-nine markers were placed on the head, shoulders, elbows, wrists, hands, upper arms, forearms, the right scapula, C7, T10, between the clavicles, sternum, anterior and posterior superior iliac spines, thighs, tibiae, heels, and big toes^17^. Six additional markers were placed on the knees, ankles and pinky toes (Fig. 1c). Initial data reconstruction, labeling and filtering as well as identifying temporal events (e.g., reaching maximal vertical height during DVJ activities) were implemented using Nexus (version 2.6, Vicon UK, Oxford, UK). Segmental rotations and joint coordinates were estimated using an in-house code. Marker locations were used as input to the musculoskeletal model (Sect. Musculoskeletal modeling study) to estimate biomechanical loads on the spine during gait and DVJ activities.

#### Force-plate

GRF and CoP data were recorded simultaneously using two adjacent force platforms (Kistler Instrument AG, Switzerland) at a sampling rate of 1200 Hz. Force-plate data were low-pass filtered at 10 Hz, using a dual-pass 4th order Butterworth filter. GRF and CoP data were also used as input into the biomechanical model (Sect. Musculoskeletal modeling study). Figures A.1 and A.2 in Appendix A indicate GRF during DVJ and CoP during FL phase of DVJ for a subject, respectively, as the samples of experimental data recorded.

**Figure 2:**
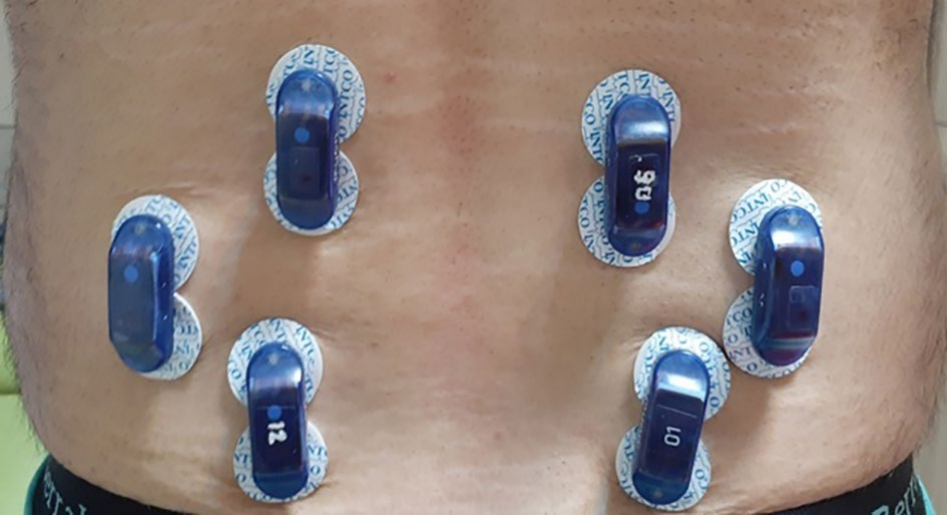
Placement of EMG electrodes on back muscles.

#### EMG collection

In order to investigate the effect of footwear conditions on lumbosacral compression force, EMG activities of main trunk extensor and flexor muscles were recorded^3^. EMG activities of left/right longissimus (LG-L/LG-R), iliocostalis (IC-L/IC-R), multifidus (MF-L/MF-R), and rectus abdominis (RA-L/RA-R) muscles were collected using an eight-channel wireless system at a sampling rate of 1200 Hz (Myon 320, Switzerland). Excessive hairs that may occlude a muscle site were shaved and a brisk wipe using an alcohol swab was used to clean the skin. Conductive electrode paste was subsequently applied on the center of electrodes to ensure a firm placement. Bipolar surface electrodes were then placed over the target muscles and parallel to the muscle fibers at: ~ 4 cm lateral to the L1 spinous process for LG, ~ 6 cm lateral to the L2 for IC, ~ 2 cm lateral to the L5 for MF, and ~ 2 cm lateral to the umbilicus for RA^18,19^ (Fig. 2). EMG raw data were high pass filtered at 35 Hz, demeaned, rectified, and low-pass filtered at 40 Hz^20^. The high pass filter was used to attenuate low-frequency noises and make the data sharpen with an improved quality^4^. A 50-Hz notch filter was also applied to eliminate the power line noise. All EMG recordings were visually inspected for any noise spike^21^. For each of the selected muscles, distinct maximal voluntary isometric contraction (MVIC) test was carried out. For erector spinae muscles, for instance, the MVIC was measured during maximal trunk extension against resistance in the horizontal position, known as the Biering–Sorensen maneuver^5^. EMG data were normalized to the measured maximum activities of each muscle^22^ and average integrated EMGs (aIEMG) were calculated^23^. Motion, EMG, and force-plate data were synchronized. EMG data were also used to validate the biomechanical model.

#### Tasks

In DVJ activity, the subject stepped off a height of 35 cm and landed with both feet onto the two adjacent force plates; i.e., each foot was completely placed on the corresponding force plate (first landing (FL) phase). Subsequently, the subject immediately jumped as high as possible and landed again on the same force plates (second landing (SL) phase)^4^. FL phase begins when the initial contact between feet and ground occurs and finishes when subject takes off for the jump. SL phase starts when subject lands after the jump and finishes when subject rests in the standing position^4^. During the entire DVJ activity, arms were fully free with no restrictions. Three trials for different footwear conditions were recorded for each subject, i.e., total of nine trials for each participant to ensure the repeatability of the experimentations. A 3-min recovery time was applied between the experiments to avoid likely effects of muscles’ fatigue^9^. Moreover, each participant was asked to perform a gait cycle. Participants walked at self-selected speed over two force plates (one force plate for each foot). Similar to DVJ experiments, three trials for different footwear conditions were recorded, i.e., total of nine trials for each participant. The intraclass correlation coefficients (ICC), calculated for all the analyzed biomechanical variables (e.g., vGRF and lumbosacral compression force), ranged from 0.85 to 0.93 thus suggesting a satisfactory repeatability of the tests. Data from different trials were input separately to the model and nine simulations were performed for each subject (i.e., three trials for three conditions). The mean value of each model output was subsequently used for the statistical analyses.

### Musculoskeletal modeling study

To model DVJ and gait activities, OpenSim, an open-source musculoskeletal modeling software, was utilized. Among all the available full body models, FBLS model consisting of 21 segments, 30 degrees of freedom, and 324 musculotendon actuators was used^24^. The generic musculoskeletal model was scaled to match each individual’s anthropometry^25^. Motion and force-plate data were input into the model that predicted muscle forces and spine loads. Motion and mass properties of the model were optimized using inverse kinematics and residual reduction algorithms to achieve a dynamically consistent set of kinematics and kinetics that best matched the experimentally collected data^25^. Subsequently, a static optimization algorithm, that minimized sum of cubed muscle activations, was applied to resolve the net moments of joints into individual muscle forces at each instant in time^26^. Finally, reaction forces for each joint were determined using the analyze tool in the software. Muscle activities, lumbosacral (L5-S1) compressive and shear loads as well as L5-S1 external and passive moments were estimated^26^ during each activity and footwear condition.

### Statistical analyses

After performing experiments and simulations, for each parameter the mean value of the three trials was used per individual in different footwear conditions. Therefore, with 11 subjects and three footwear conditions, total of 33 data were used for the statistical analyses. Repeated measure ANOVAs were employed to investigate effects of test conditions; e.g., to verify whether different shod conditions affect the maximum vGRF during the FL of DVJ. The normality of the data was examined by performing the Shapiro–Wilk test. For all biomechanical variables, the Shapiro–Wilk *p*-value was larger than 0.05 implying that data follow normal distributions. Tukey’s honestly significant difference (HSD) tests were carried out when the results of ANOVA showed a significant difference between various footwear conditions. For all statistical analyses, IBM SPSS Statistics for Windows (Version 26.0. Armonk, NY: IBM) was used. To validate the biomechanical model, Pearson correlation coefficient (r) was calculated between the predicted muscle activations and measured EMG data.

## Results

**Figure 3:**
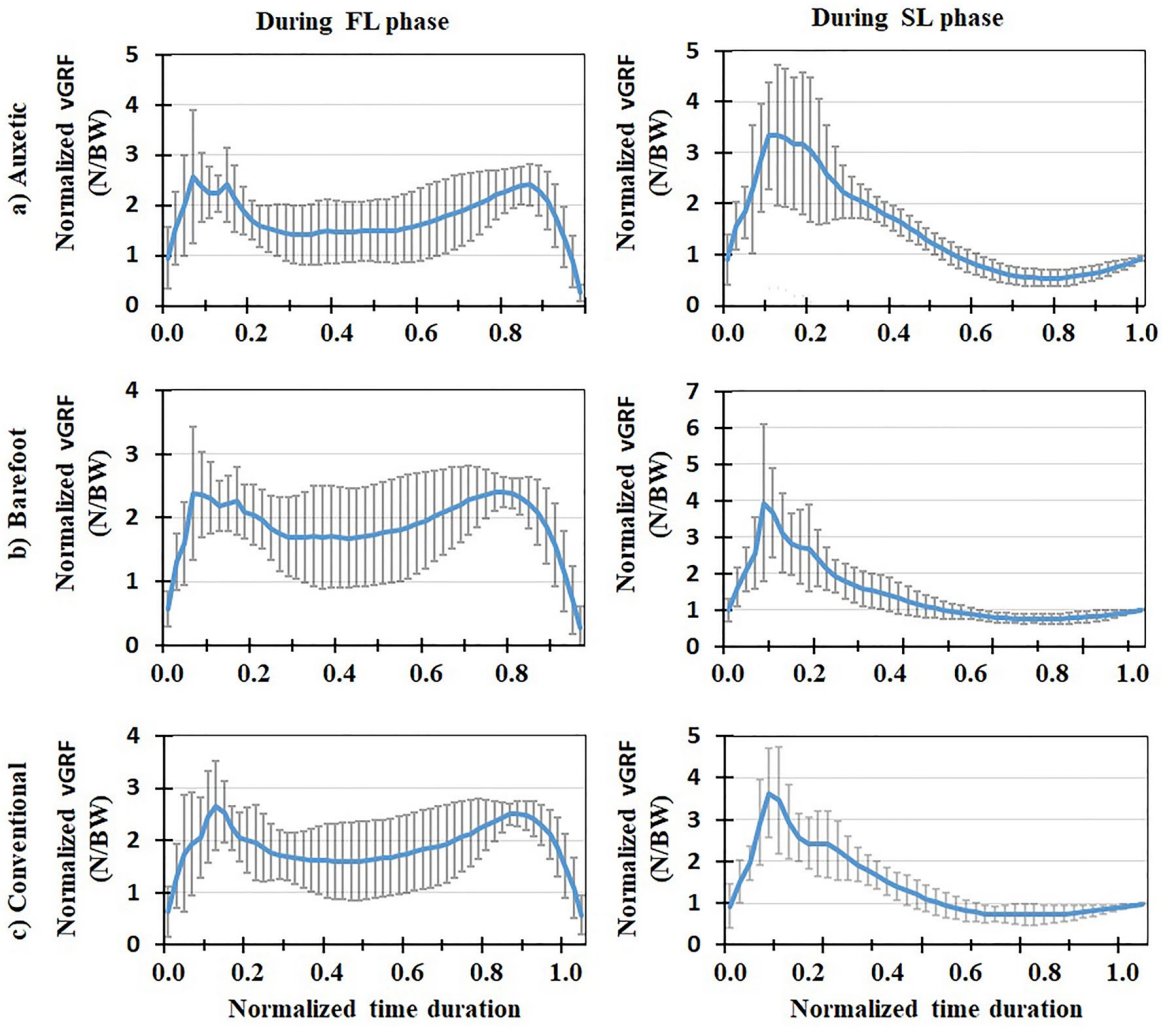
Mean normalized vGRF (N/BW) for all the participants (+ / − one standard deviation) in three footwear conditions: (**a**) auxetic shoe, (**b**) barefoot, and (**c**) conventional shoe. Left and right plots indicate variations during FL and SL phases of DVJ, respectively.

**Figure 4:**
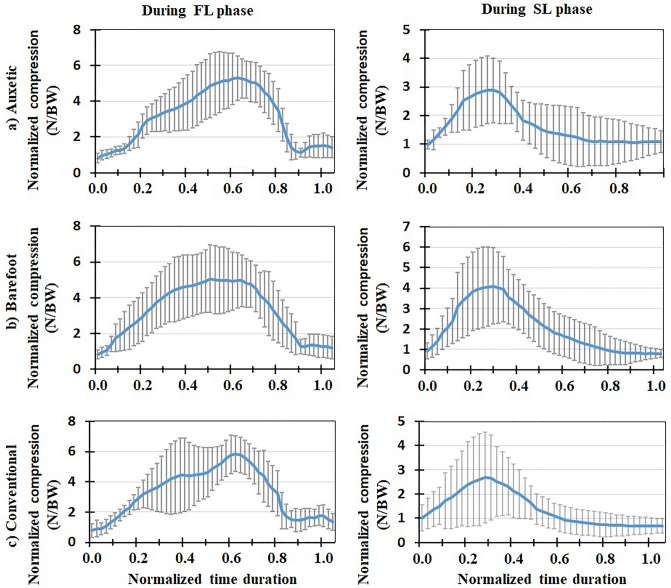
Mean normalized lumbosacral (L5-S1) compression force (N/BW) for all the participants (+ / − one standard deviation) in three footwear conditions: (**a**) auxetic shoe, (**b**) barefoot, and (**c**) conventional shoe. Left and right plots indicate variations during FL and SL phases of DVJ, respectively.

**Figure 5:**
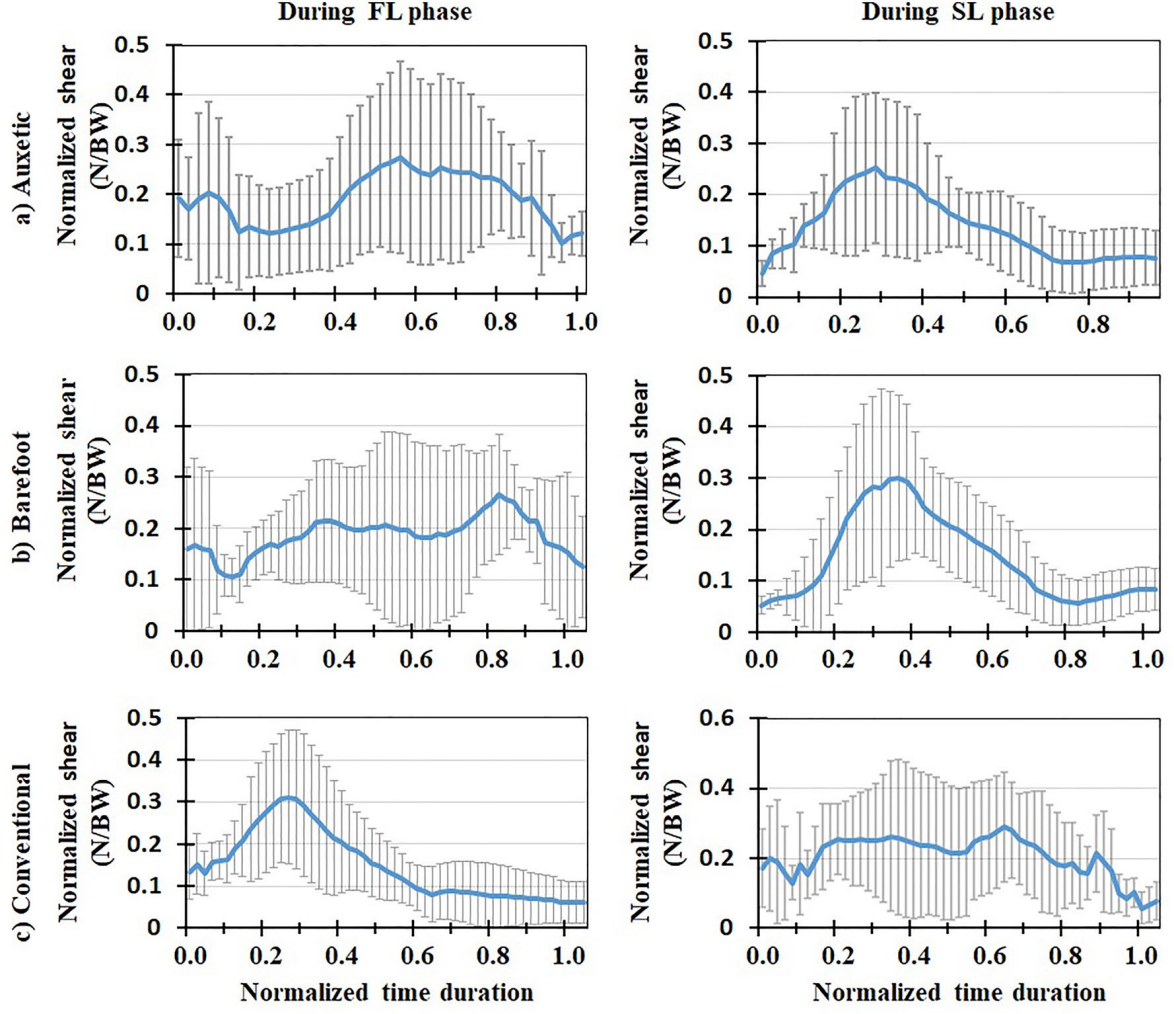
Mean normalized lumbosacral (L5-S1) shear force (N/BW) for all the participants (+ / − one standard deviation) in three footwear conditions: (**a**) auxetic shoe, (**b**) barefoot, and (**c**) conventional shoe. Left and right plots indicate variations during FL and SL phases of DVJ, respectively.

**Figure 6:**
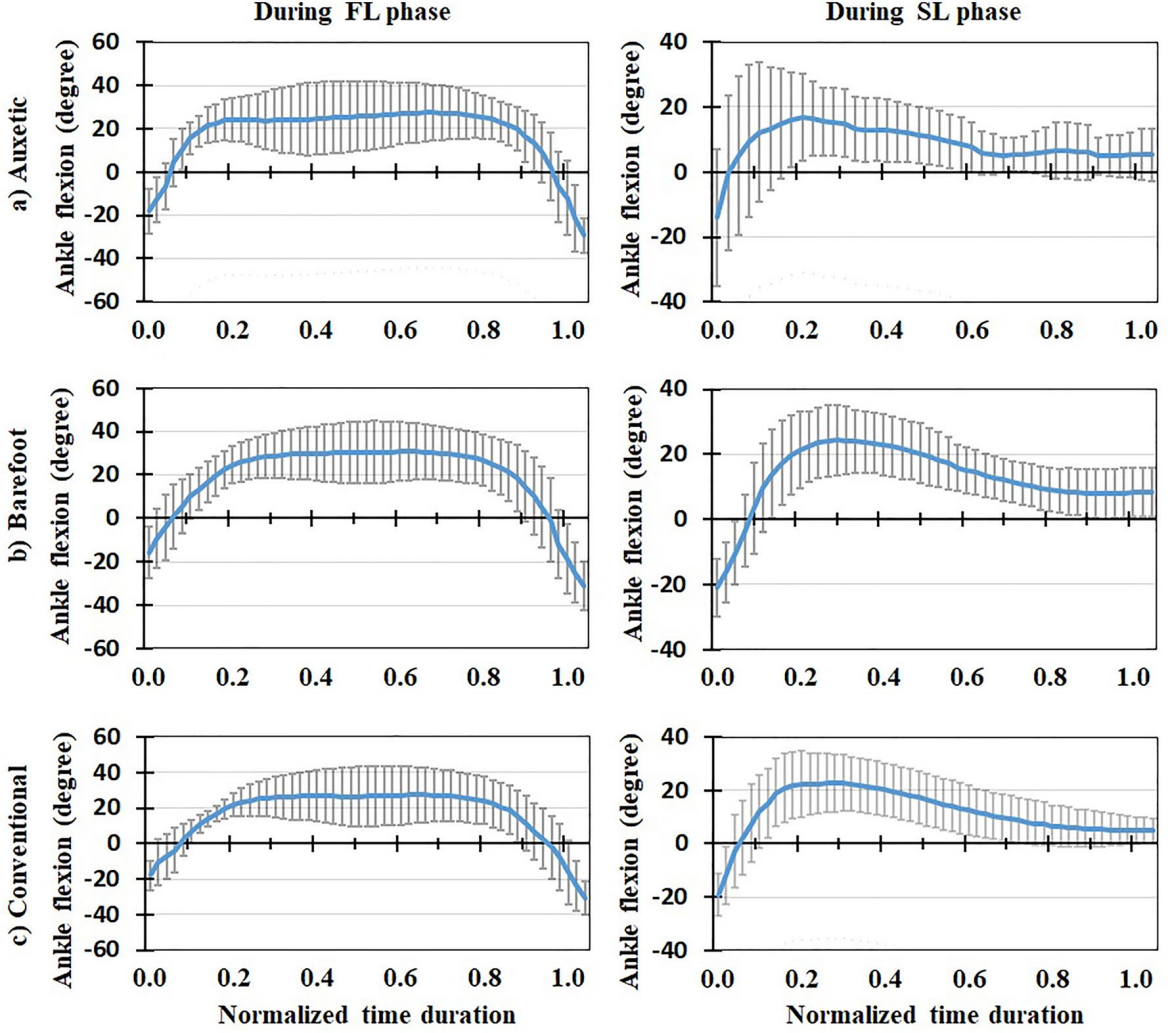
Mean Ankle flexion (degree) for all the participants (+ / − one standard deviation) in three footwear conditions: (**a**) auxetic shoe, (**b**) barefoot, and (**c**) conventional shoe. Left and right plots indicate variations during FL and SL phases of DVJ, respectively.

**Figure 7:**
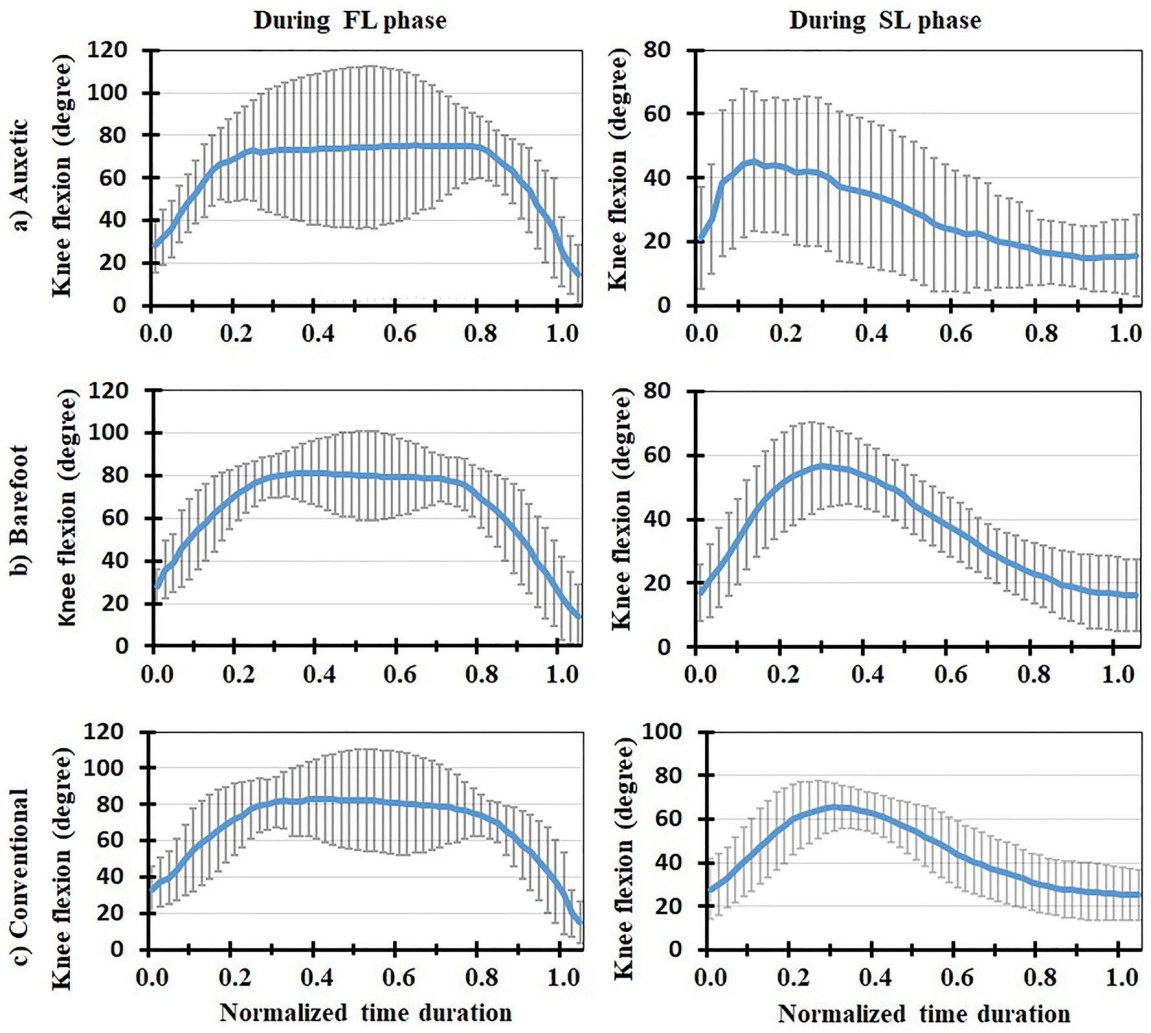
Mean knee flexion (degree) for all the participants (+ / − one standard deviation) in three footwear conditions: (**a**) auxetic shoe, (**b**) barefoot, and (**c**) conventional shoe. Left and right plots indicate variations during FL and SL phases of DVJ, respectively.

**Figure 8:**
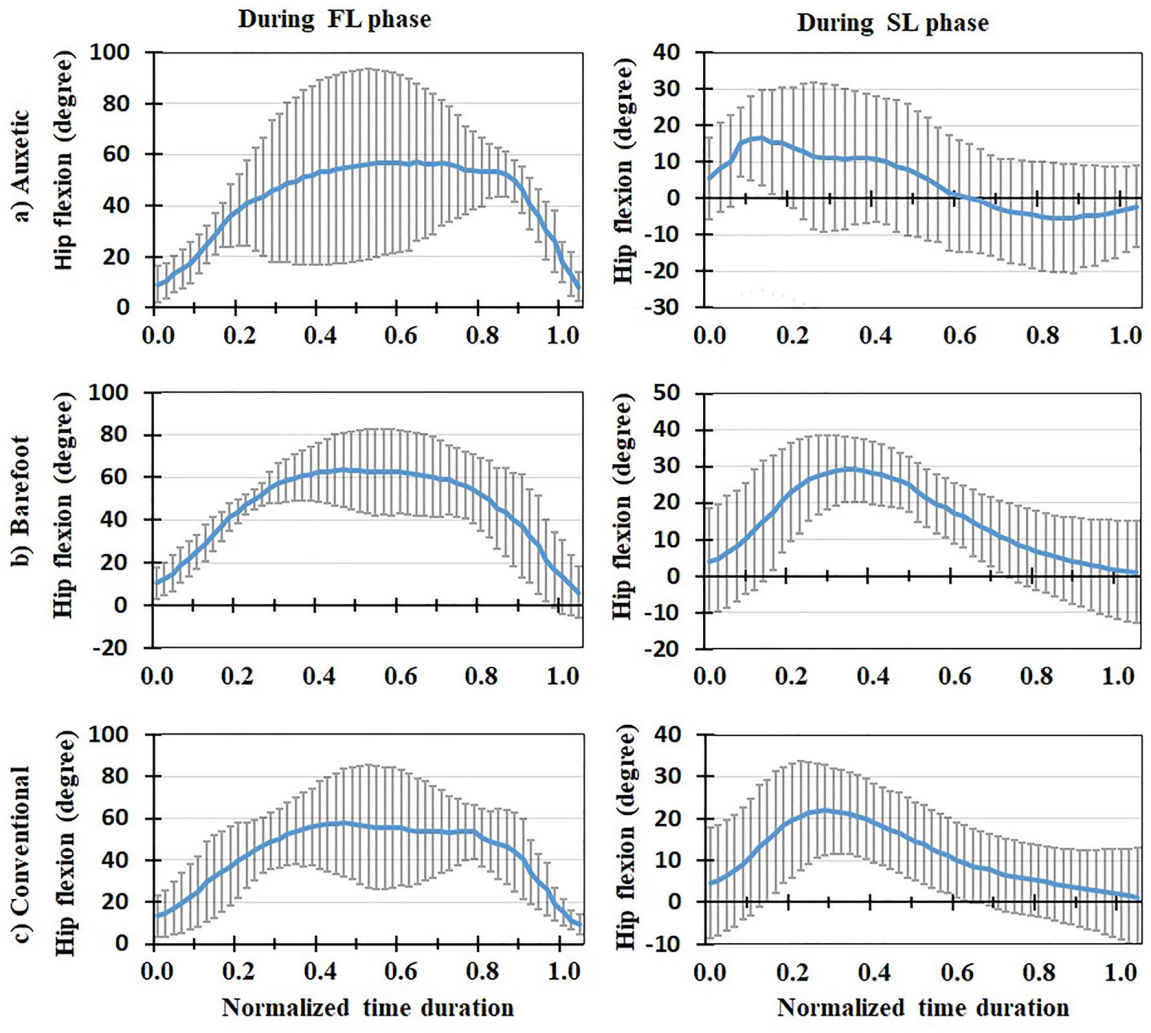
Mean hip flexion (degree) for all the participants (+ / − one standard deviation) in three footwear conditions: (**a**) auxetic shoe, (**b**) barefoot, and (**c**) conventional shoe. Left and right plots indicate variations during FL and SL phases of DVJ, respectively.

**Figure 9:**
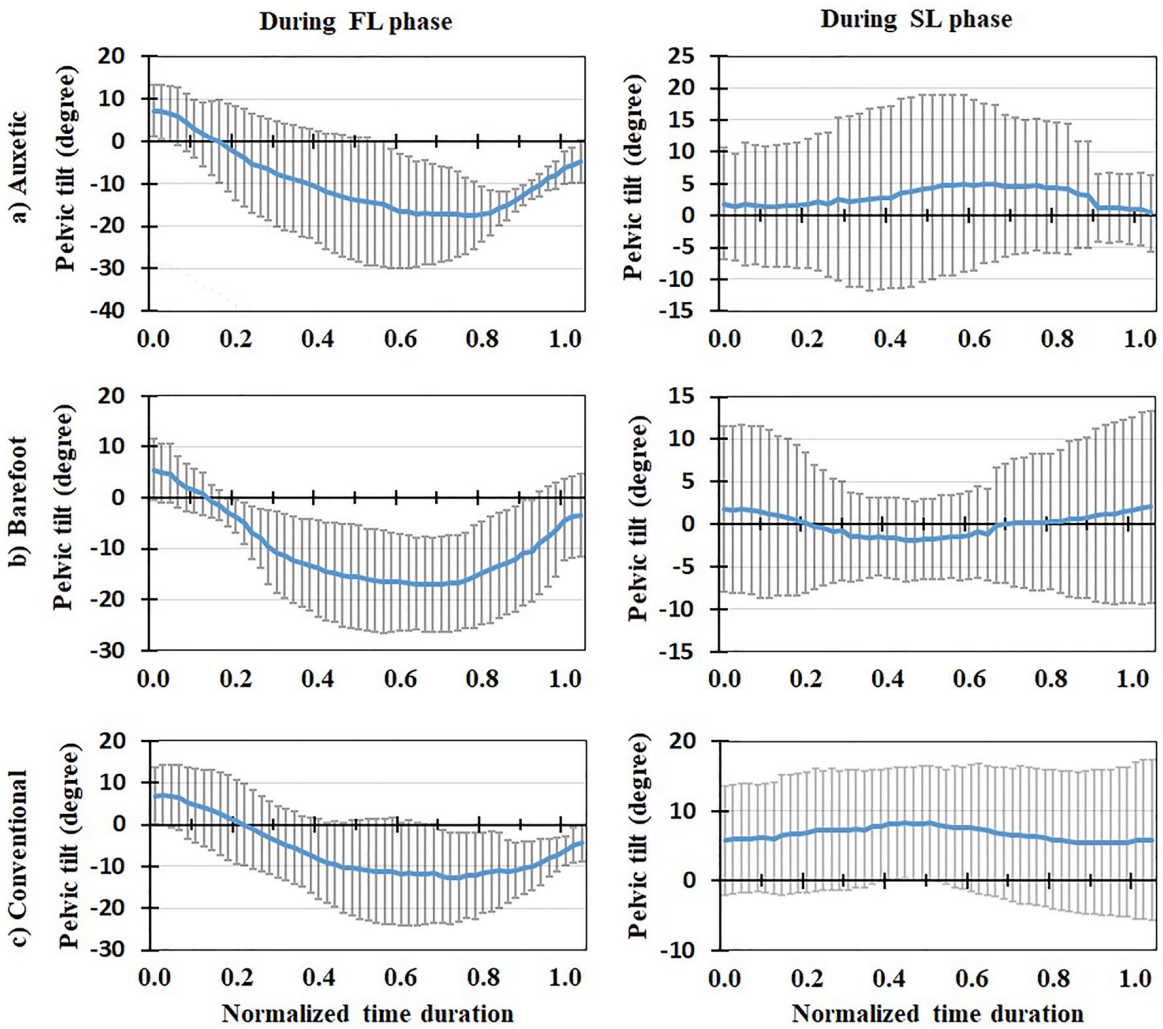
Mean pelvic tilt (degree) for all the participants (+ / − one standard deviation) in three footwear conditions: (**a**) auxetic shoe, (**b**) barefoot, and (**c**) conventional shoe. Left and right plots indicate variations during FL and SL phases of DVJ, respectively.

**Figure 10:**
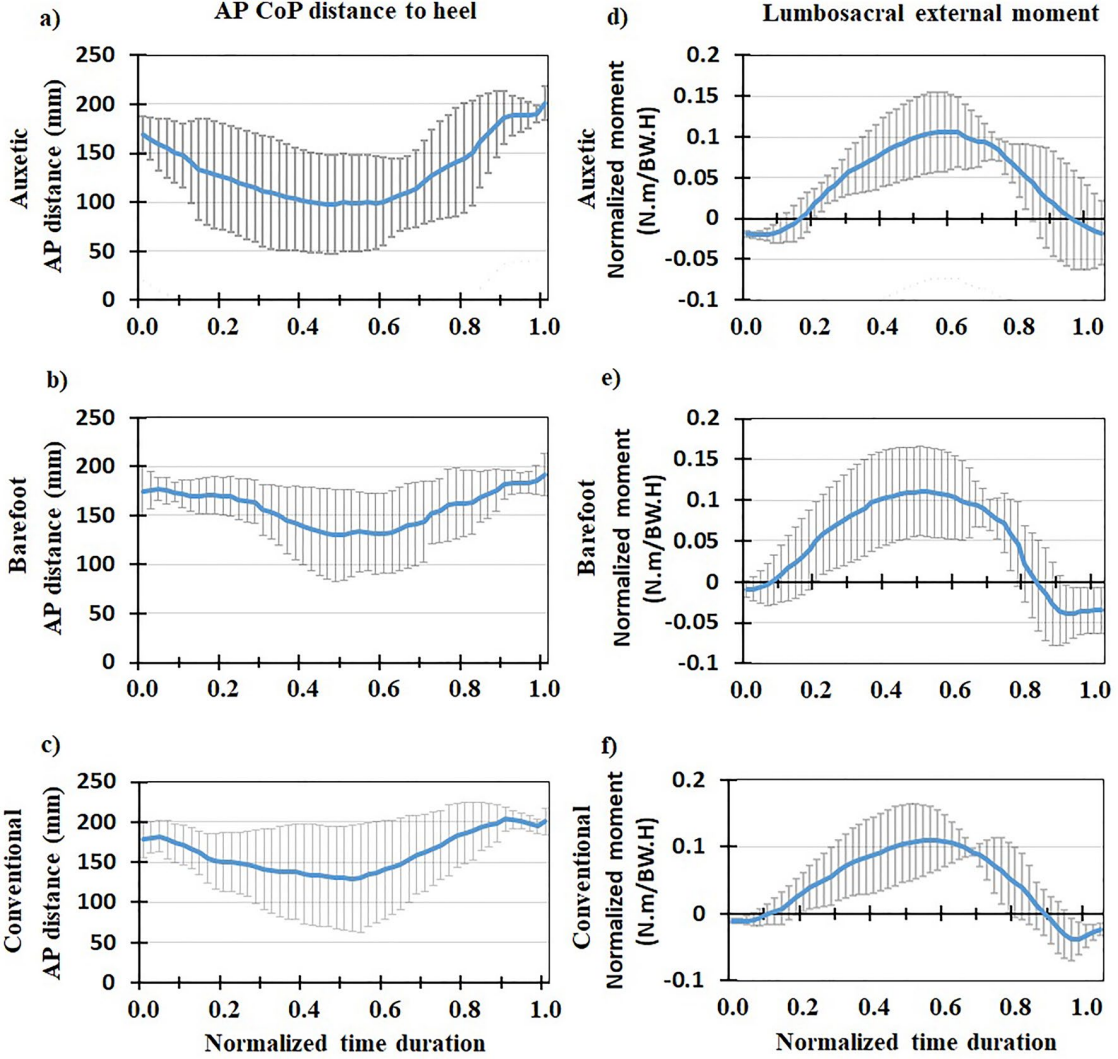
(Left) AP distance between CoP of GRF and heel (mm) (+ / − one standard deviation) during the FL phase of DVJ in three footwear conditions: (**a**) auxetic shoe, (**b**) barefoot, and (**c**) conventional shoe. (Right) mean normalized lumbosacral external moment (N m/BW H) (+ / − one standard deviation) during the FL phase of DVJ in three footwear conditions: (**d**) auxetic shoe, (**e**) barefoot, and (**f**) conventional shoe.

Mean (+ / − one standard deviation) values (of all the subjects) of biomechanical variables during both FL and SL phases of DVJ were plotted after normalizing time durations between 0 and 1 (Figs. 3, 4, 5, 6, 7, 8, 9, 10). The calculated forces (i.e., vGRF and lumbosacral compression/shear forces) were normalized to subject’s body weight (Figs. 3, 4, 5). The lumbosacral external flexor moment was normalized to subject’s body weight times body height (Fig. 10d–f). As our objective was to examine the flexor moment (in the sagittal plane) generated by GRF at the L5-S1 joint, only the AP distance of CoP to the heel was considered in our calculations. The latter variable was not normalized as all subjects were using the same shoe in each shoe condition examined.

The CoP and heel position were determined by the force plate and markers, respectively. To find the AP distance between the heel and CoP, a plane parallel to the sagittal plane that passes through the heel and hallux marker was defined. AP distance of CoP to heel was the projection of the vector from the heel to CoP on the foregoing plane. For each trial, two CoP values, one for the right foot and one for the left foot, were measured and separately input into the model. Results indicated that shoe condition had no effect on none of the model predicted or in vivo measured data during the gait cycle and SL phase of DVJ. Results are, therefore, reported hereafter for the FL phase of DVJ:

### In vivo measured EMG, kinematics and force-plate data

During the FL phase, EMG activities of longissimus and iliocostalis muscles were significantly smaller (*p* < 0.001) in auxetic shoe condition as compared to both conventional (by ~ 29 and 16%, respectively) and barefoot (by ~ 37 and 19%, respectively) conditions (Table 1). Shoe condition had no significant effect on EMG activities of multifidus and rectus abdominis muscles, maximal vGRF, and minimum pelvic tilt (Table 1). AP distance between the CoP and heel decreased to a minimal value and subsequently increased to approximately its initial value (Fig. 10). The minimal value of the AP distance between the CoP and heel occurred when the ankle, knee, and hip flexions were maximized and pelvic tilt was minimized (Figs. 6, 7, 8, 9), i.e., when the center of gravity of the subject was in its lowest height and the subject was ready for the jumping. The AP distance between the CoP and heel was significantly smaller in auxetic shoe condition (*p* = 0.002) as compared to both conventional (by ~ 19%) and barefoot (by ~ 20%) conditions (Table 1). Maximal hip, knee, and ankle flexion angles were generally larger (*p* < 0.005) in auxetic shoe condition as compared to both conventional and barefoot conditions (Table 1).

**Table 1:** Effects of different footwear conditions on various biomechanical parameters during the FL phase of DVJ based on ANOVA and Tukey’s tests.

Biomechanical investigated variable	*p*-value	Significant difference (*p*-value < 0.05)?	Tukey’s HSD test results		Mean values in different conditions		
			Conventional-auxetic	Barefoot-auxetic	Conventional	Barefoot	Auxetic
Lumbosacral maximum compression force	< 0.001	✓	11.8%	8.2%	6.56 N/BW	6.29 N/BW	5.77 N/BW
Lumbosacral average shear force	0.607	✗	No significant difference		0.215 N/BW	0.243 N/BW	0.238 N/BW
Lumbosacral maximum external moment	0.022	✓	8.1%	8.3%	0.175 N.m/BW.H	0.175 N.m/BW.H	0.161 N.m/BW.H
Lumbosacral maximum passive moment	0.822	✗	No significant difference		0.054 N.m/BW.H	0.048 N.m/BW.H	0.052 N.m/BW.H
Longissimus aIEMG	< 0.001	✓	29.1%	37.2%	0.272%MVIC	0.305%MVIC	0.193%MVIC
Iliocostalis aIEMG	< 0.001	✓	16.1%	18.8%	0.515%MVIC	0.532%MVIC	0.432%MVIC
Rectus abdominis aIEMG	0.341	✗	No significant difference		0.432%MVIC	0.398%MVIC	0.425%MVIC
Multifidus aIEMG	0.121	✗	No significant difference		0.542%MVIC	0.584%MVIC	0.498%MVIC
Maximum vertical ground reaction force	0.09	✗	No significant difference		2.58 N/BW	2.51 N/BW	2.59 N/BW
Minimum anterior–posterior distance from GRF CoP to heel	0.002	✓	19.1%	19.9%	138.5 mm	137.2 mm	111.0 mm
Minimum pelvic tilt	0.277	✗	No significant difference		− 16.5°	− 17.8°	− 15.9°
Maximum Hip flexion	0.001	✓	− 13.4%	− 12.2%	72.6°	73.3°	82.3°
Maximum knee flextion	0.005	✓	− 9.0%	− 4.2%	96.2°	92.2°	100.3°
Maximum ankle flexion	0.002	✓	− 13.2%	− 11.61%	29.8°	30.2°	33.7°

### Model predictions for muscle activities and lumbosacral loads

Predicted muscle activities were in good/excellent agreements to measured EMGs (Table 2); Pearson correlation coefficients (r values) ranged from 0.68 to 0.88. During the FL phase, lumbosacral (L5-S1) compression load and external moment increased to a maximal value and subsequently decreased to its initial value (Figs. 4 and 10). The maximal values of load/moment occurred approximately at the same time; i.e., when the center of gravity of the subject was at its lowest height (Figs. 6, 7, 8, 9). The maximal L5-S1 compression force and maximal external moment were significantly smaller (*p* < 0.001) in auxetic shoe condition as compared to both conventional (by ~ 12 and 8%, respectively) and barefoot (by ~ 8 and 8%, respectively) conditions (Table 1). Shoe condition had no significant effect on the shear and passive (resistive) moment of the L5-S1 joint (Table 1).

**Table 2:** Pearson correlation coefficients (r) calculated between measured (EMG) and model predicted muscle activations during the FL phase of DVJ for different trunk muscles.

Muscle name	Calculated r
Longissimus	0.758 ± 0.014
Iliocostalis	0.748 ± 0.025
Rectus Abdominis	0.883 ± 0.026
Multifidus	0.683 ± 0.023
All the 4 muscles	0.768 ± 0.026

## Discussion

This is the first study to our knowledge that investigates the effects of auxetic shoes on the biomechanics of the spine. The main objective was to compare auxetic and conventional shoes in terms of lumber spine kinematics and kinetics during gait and DVJ. While no significant changes were observed in gait and the SL phase of DVJ, results showed significant differences between footwear conditions in the FL phase of DVJ; i.e., wearing the auxetic shoe caused the following outcomes: (a) smaller EMG activities in longissimus and iliocostalis muscles, (b) smaller AP distance between the CoP and heel, (c) larger maximal hip, knee, and ankle flexion angles and finally (d) smaller maximal L5-S1 compression force and maximal external moment. These outcomes confirm our hypothesis on the beneficial effect of wearing auxetic shoes on spine biomechanics during DVJ^11,15^.

It is believed that insignificant changes between the three shoe conditions during our gait experiment was due to the low biomechanical response of body during light activities. This independence of biomechanical reaction on shoe condition was also observed in the SL phase of DVJ where the subject was asked to gently drop on the force-plate without any following activity. A similar outcome was observed by another study^13^ where the changes in the kinematics and kinetics were found insignificant during barbell back squat experiments in three shoe conditions; i.e., barefoot, running shoes, and weightlifting shoes. In their study, the body was not subject to quick movements and impacts either.

The variations of biomechanical parameters with footwear conditions in the FL phase of DVJ, however, can be associated with an important difference of this phase with both gait and SL phase of DVJ in terms of intensity of activity and velocity of motion. That is, as necessary in the FL phase of DVJ, the subject jumped up with the maximum power once his feet fully touched the ground. Using auxetic shoes was found to render a reduction in the minimum AP distance between the CoP of GRF and heel compared to other two footwear conditions. The reduction in this distance, as the moment arm of GRF, decreased the required external flexor moment at the lumbosacral joint, hence reducing the activation of extensor muscles (i.e., longissimus and iliocostalis) which, in turn, decreased the lumbosacral compression force. Dependence of kinetic and kinematic parameters on footwear condition during the FL phase of DVJ activity has also been reported in the literature; e.g., larger ankle joint angles at initial ground contact, smaller knee joint angles between the second peak GRF and take-off as well as smaller foot strike angles at both initial ground contact and take-off when wearing shoes as compared to barefoot conditions^9^. Moreover, the shoes with a softer midsole have been found to generate a higher forefoot peak force amid a lower rearfoot peak force, lower peak flexion moments at the ankle and hip joints, and greater prelanding muscle activations in the rectus femoris and tibialis anterior^11^. We may hence conclude that the effects of auxetic shoes are more pronounced during more demanding activities where heavy body reactions are required. This is consistent with our expectation from an auxetic structure to cause superior energy absorption in shock/impact loading conditions compared to conventional foams as reported elsewhere^15^.

In this study, we attempted to eliminate effects of inter-individual differences caused by individuals’ performances and their physical variabilities. The shoes’ parameters, however, were not isolated; i.e., the authors acknowledge the fact that the reported differences between the shoe conditions in the FL phase of DVJ activity may not be explicitly attributed to a specific characteristic such as midsole material and/or structure, outsole flexibility, or shoe sole profile. Moreover, as the effect of shoe conditions was found to be task-dependent, it remains to correlate kinematics and kinetics of a given activity to the role of shoe condition in biomechanical response of body. To control the effect of confounding parameters such as gender and age, only young male individuals participated in the present study. To investigate the distinct effects of sex and age on the beneficial role of auxetic shoes, much more subjects are required to take part in the in vivo tests while additional efforts are also needed for our subject-specific modeling study.

In conclusion, three different footwear conditions were examined using a combined biomechanical in vivo*-*modelling experimentation during gait and DVJ activities. Motion, EMG, and force data were obtained from experiments and a musculoskeletal model was developed and validated using the measured EMGs. The model was used to predict the L5-S1 compressive and shear loads as well as external and passive moments. No significant differences were found between barefoot, conventional shoe and auxetic shoe conditions during the gait and SL phase of DVJ. In the course of FL phase of DVJ, however, auxetic shoes generated smaller EMG activities in longissimus and iliocostalis muscles, smaller AP distance between the CoP and heel, larger maximal hip, knee, and ankle flexion angles, and smaller maximal L5-S1 compression force and maximal external moment. Hence, the effect of auxetic shoes was found discernible during the tasks associated with quick movements and high impacts. In other words, our findings indicate that wearing auxetic shoes will lead to a lower short-term compression force on the lumbar spine during high-demanding activities. This may reduce the risk of low back pain/disorder in long term for individuals dealing with such activities. Further investigations are required to discriminate the effects of different parameters of shoe sole on lumber spine kinematics and kinetics.

## Supplementary Information

Supplementary Legends.

Supplementary Information 2.

Supplementary Information 3.

## References

[1] Michalik R (2019). Comparison of two different designs of forefoot off-loader shoes and their influence on gait and spinal posture. Gait Posture.

[2] MacRae CS, Critchley D, Morrissey M, Shortland A, Lewis JS (2016). Do rocker-sole shoes influence postural stability in chronic low back pain? A randomised trial. BMJ Open Sports Exerc. Med..

[3] Khoury-Mireb M, Solomonow-Avnon D, Rozen N, Wolf A (2019). The effect of unstable shoe designs on the variability of gait measures. Gait Posture.

[4] Bates NA, Ford KR, Myer GD, Hewett TE (2013). Impact differences in ground reaction force and center of mass between the first and second landing phases of a drop vertical jump and their implications for injury risk assessment. J. Biomech..

[5] Simpson JD, Miller BL, O’Neal EK, Chander H, Knight AC (2018). Ground reaction forces during a drop vertical jump: Impact of external load training. Hum. Mov. Sci..

[6] Collin Herb, C., Grossman, K., Feger, M. A., Donovan, L. & Hertel, J. Lower extremity biomechanics during a drop-vertical jump in participants with or without chronic ankle instability. *J. Athl. Train.* **53**, 364–371 (2018). 10.4085/1062-6050-481-15 PMC5967278 29667844

[7] Čoh M (2015). Kinematic, dynamic and EMG analysis of drop jumps in female elite triple jump athletes. Coll. Antropol..

[8] Peng HT, Kernozek TW, Song CY (2011). Quadricep and hamstring activation during drop jumps with changes in drop height. Phys. Ther. Sport.

[9] Koyama K, Yamauchi J (2018). Comparison of lower limb kinetics, kinematics and muscle activation during drop jumping under shod and barefoot conditions. J. Biomech..

[10] Fong Yan, A., Hiller, C., Sinclair, P. J. & Smith, R. M. Kinematic analysis of Sautés in barefoot and shod conditions. *J. Dance Med. Sci.* **18**, 149–158 (2014). 10.12678/1089-313X.18.4.149 25474294

[11] Alonzo R (2020). Effects of basketball shoe midsole hardness on lower extremity biomechanics and perception during drop jumping from different heights. Appl. Sci. (Switzerland).

[12] Boyer KA, Andriacchi TP (2009). Changes in running kinematics and kinetics in response to a rockered shoe intervention. Clin. Biomech..

[13] Southwell DJ, Petersen SA, Beach TAC, Graham RB (2016). The effects of squatting footwear on three-dimensional lower limb and spine kinetics. J. Electromyogr. Kinesiol..

[14] Sanami M, Ravirala N, Alderson K, Alderson A (2014). Auxetic materials for sports applications. Procedia Eng..

[15] Moroney, C., Alderson, A., Allen, T., Sanami, M. & Venkatraman, P. The application of auxetic material for protective sports apparel. *Proceedings* **2**, 251 (2018).

[16] M. Cross, T., W. Hoffer, K., P. Jones, D., B. Kirschner, E. & C. Meschter, J. Auxetic structures and footwear with soles having auxetic structures. vol. 2 402–439 (2016).

[17] Asadi F, Arjmand N (2020). Marker-less versus marker-based driven musculoskeletal models of the spine during static load-handling activities. J. Biomech..

[18] Haddad, O. *Development and validation of a fatigue-modified, EMG-assisted biomechanical model of the lumbar region*. (2011).

[19] Haddad O, Mirka GA (2013). Trunk muscle fatigue and its implications in EMG-assisted biomechanical modeling. Int. J. Ind. Ergon..

[20] Chvatal SA, Torres-Oviedo G, Safavynia SA, Ting LH (2011). Common muscle synergies for control of center of mass and force in nonstepping and stepping postural behaviors. J. Neurophysiol..

[21] Trask C (2010). EMG estimated mean, peak, and cumulative spinal compression of workers in five heavy industries. Int. J. Ind. Ergon..

[22] Alenabi T, Whittaker R, Kim SY, Dickerson CR (2018). Maximal voluntary isometric contraction tests for normalizing electromyographic data from different regions of supraspinatus and infraspinatus muscles: Identifying reliable combinations. J. Electromyogr. Kinesiol..

[23] Abdoli-E M, Stevenson JM (2008). The effect of on-body lift assistive device on the lumbar 3D dynamic moments and EMG during asymmetric freestyle lifting. Clin. Biomech..

[24] Raabe ME, Chaudhari AMW (2016). An investigation of jogging biomechanics using the full-body lumbar spine model: Model development and validation. J. Biomech..

[25] Delp SL (2007). OpenSim: Open-source software to create and analyze dynamic simulations of movement. IEEE Trans. Biomed. Eng..

[26] Steele KM, DeMers MS, Schwartz MH, Delp SL (2011). Compressive tibiofemoral force during crouch gait. Gait Posture.

