# Supplementary material for: Effects of auxetic shoe on lumbar spine kinematics and kinetics during gait and drop vertical jump by a combined in vivo and modeling investigation
Source: Sci Rep. 2022 Oct 31;12:18326. doi: 10.1038/s41598-022-21540-6 (PMC9622817; doi:10.1038/s41598-022-21540-6)
Supplement: Supplementary file 1 — Supplementary Legends. [file 41598_2022_21540_MOESM1_ESM.docx]

**Appendix A.**

Figures A.1 and A.2 indicate GRF during DVJ and CoP during FL phase of DVJ for a subject, respectively, as the samples of experimental data recorded.

**Figure 1A:** vGRF of a subject during DVJ. The first and second peaks represent the FL and SL phases, respectively.

**Figure 2A:** AP CoP distance to the heel of a subject during FL of DVJ.
