# Supplementary figures and images for: Effects of auxetic shoe on lumbar spine kinematics and kinetics during gait and drop vertical jump by a combined in vivo and modeling investigation
Source: Sci Rep. 2022 Oct 31;12:18326. doi: 10.1038/s41598-022-21540-6 (PMC9622817; doi:10.1038/s41598-022-21540-6)

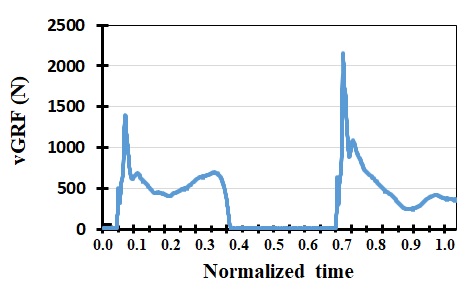

Supplement: Supplementary file 2 — Supplementary Information 2. [file 41598_2022_21540_MOESM2_ESM.jpg]

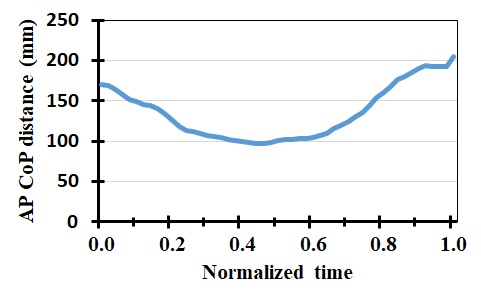

Supplement: Supplementary file 3 — Supplementary Information 3. [file 41598_2022_21540_MOESM3_ESM.jpg]
